# A model-based analysis: what potential could there be for a *S. aureus* vaccine in a hospital setting on top of other preventative measures?

**DOI:** 10.1186/1471-2334-14-291

**Published:** 2014-05-28

**Authors:** Cosmina Hogea, Thierry Van Effelterre, Adrian Cassidy

**Affiliations:** 1 GlaxoSmithKline Vaccines, 2301 Renaissance Blvd Ste RN0510, King of Prussia, PA, USA; 2 GlaxoSmithKline Vaccines, Wavre, Belgium

**Keywords:** Mathematical modelling, MRSA, Hospital infection, Infection reduction, Vaccination, Preventative measures

## Abstract

**Background:**

Over the past decade, there has been sustained interest and efforts to develop a *S. aureus* vaccine. There is a need to better evaluate the potential public health impact of *S. aureus* vaccination, particularly given that preventative measures exist to reduce infection. To our knowledge, there is no previous work to assess the potential of a *S. aureus* vaccine to yield additional MRSA infection reduction in a hospital setting, on top of other preventative measures that already proved efficient.

**Methods:**

The main objectives were to propose a versatile simulation framework for assessing potential added benefits of a hypothetical *S. Aureus* vaccine in conjunction with other preventative measures, and to illustrate possibilities in a given hospital setting. To this end, we employed a recently published dynamic transmission modelling framework that we further adapted and expanded to include a hypothetical *S. aureus* vaccination component in order to estimate potential benefits of vaccinating patients prior to hospital admission.

**Results:**

Model-based projections indicate that even with other hygiene prevention measures in place, vaccination of patients prior to hospital admission has the potential to provide additional reduction of MRSA infection. Vaccine coverage and vaccine efficacy are key factors that would ultimately impact the magnitude of this reduction. For example, in an average case scenario with 50% decolonization, 50% screening and 50% hygiene compliance level in place, *S. aureus* vaccination with 25% vaccine coverage, 75% vaccine efficacy against infection, and 0% vaccine efficacy against colonization, may lead to 12% model-projected additional reduction in MRSA infection prevalence due to vaccination, while this reduction could reach 37% for vaccination with 75% vaccine coverage and 75% vaccine efficacy against infection in the same average case scenario.

**Conclusions:**

*S. aureus* vaccination could potentially provide additional reduction of MRSA infection in a hospital setting, on top of reductions from hygiene prevention measures. The magnitude of such additional reductions can vary significantly depending on the level of hygiene prevention measures in place, as well as key vaccine factors such as coverage and efficacy. Identifying appropriate combinations of preventative measures may lead to optimal strategies to effectively reduce MRSA infection in hospitals.

## Background

*Staphylococcus aureus (S. aureus)* is an important opportunistic pathogen and the aetiological agent of a wide range of infections, from mild skin and soft tissue infections to bactaeremia complicated by endocarditis, pneumonia, metastatic infections, sepsis or toxic shock syndrome [1]. *S. aureus* is an ubiquitous pathogen, part of the human microbiological flora. It persistently and asymptomatically colonizes up to 20-30% of humans and intermittently colonizes 50-60% [1].

Following the introduction of methicillin in 1959, various methicillin-resistant *S. aureus* (MRSA) clones (resistant to all currently available *β*-lactam antibiotics) emerged and spread worldwide [2]. Since the 1960s, when the first outbreak of healthcare-associated MRSA (HA-MRSA) was reported [3], HA-MRSA clones have been recognized as a leading cause of nosocomial infections in the United States and around the world [4,5]. A worldwide increase in the number of infections, together with the emergence of community-associated MRSA (CA-MRSA) in the late 1990s and its expansion into hospital settings [6], has increased the burden of MRSA infections [1]. Prevention of MRSA infections has become a goal for public health agencies and policy makers [7-9].

Several studies, the majority of which were conducted in individual or small groups of facilities, have recently shown that the rates of MRSA infections decreased following implementation of MRSA prevention strategies [10-15]. A number of studies have assessed the impact of using a combination of different prevention strategies (‘bundle’ measures), including universal surveillance for MRSA, contact precautions, hand hygiene and institutional culture change programs [11,12,15-18]. For example, a cluster-randomized trial evaluating the effect of active surveillance and expanded use of barrier precautions for MRSA and vancomycin-resistant *Enterococcus* (VRE) compared to ‘existing procedures’ during a 6-month intervention period, did not find that the intervention was more effective than existing practice in reducing the incidence of colonization or infection with MRSA or VRE [16]. However many studies have shown a positive effect of implementing the bundle measures in endemic settings [11,12,15,17,18]. An observational study on the effect of MRSA bundle measures, implemented in 2007 in Veterans Affairs hospitals throughout the United States, reported 62% and 45% decreases in rates of HA-MRSA infections in ICUs (Intensive Care Units) and non-ICUs following a 3-year intervention period [18].

Although the bulk of the evidence suggests that bundle measures are efficient towards reducing the incidence of MRSA nosocomial infections, the mortality, morbidity and costs associated with these infections remain high. *S. aureus* infections are the leading cause of hospitalization for surgical drainage of pus in children, of bacteremia in persons aged *>*65 years, and of prosthetic device and intravascular line infection [19]. In this context, during the last decade there has been a sustained interest and effort [19-22] to develop an effective vaccine against *S. aureus*[19], and several vaccine candidates are in early stage development [22].

Given the many challenges of developing a *S. aureus* vaccine [22], a natural timely question arises regarding its potential added value to positively impact the burden of MRSA infection at the hospital level. To address this question, we employ here a versatile dynamic transmission modelling framework complementary to, and expanding that used in D’Agata *et al.*[23], with the aim of assessing and quantifying the potential impact of a *S. aureus* vaccine in conjunction with hygiene prevention measures on reducing MRSA infection in a hospital setting. The main objectives were to both propose an adequate simulation framework for assessing potential added benefits of a hypothetical *S. aureus* vaccine in conjunction with other preventative measures and illustrate the spectrum of possibilities with a few relevant scenarios in a given hospital setting. The analyses we present here were not intended to be exhaustive, but rather to serve as an illustrative starting point.

## Methods

To our knowledge, this study is the first to evaluate if and how *S. aureus* vaccination prior to hospital admission may further reduce MRSA infection in a given hospital setting; particularly since hygiene prevention measures can be quite effective.

### Mathematical modelling overview

For our first attempt to estimate potential benefits of vaccinating patients prior to hospital admission, we chose to start by employing a recently published dynamic transmission modelling framework [23,24], which we further adapted and expanded to meet our specific objectives.

One of the main rationales for building a vaccination component on top of an existing published model [23,24] was that baseline estimates for all the model parameters were provided for a given hospital setting in the US. In line with the previously published work [23,24], the present framework is based on a dynamic transmission model for MRSA only, further split into HA-MRSA and CA-MRSA. Methicillin sensitive *S. aureus* (MSSA) is not included. The original base model structure, which explicitly accounts for HA-MRSA and CA-MRSA, with different corresponding baseline model parameters (Table 1), was preserved as actual and appropriate to account for the realities of MRSA transmission in a hospital setting in the US. The explicit inclusion of HA-MRSA and CA-MRSA is adequate here as CA-MRSA is a growing problem in US hospitals, with CA-MRSA strains genetically distinct from HA-MRSA strains and thought to have evolved separately [25]. Such features allow for potential differential transmission aspects to be taken into consideration, thus enabling more realistic estimations of the overall reduction of MRSA infection in hospital settings following implementation of different interventions. The proposed modelling framework also readily enables consideration of potentially different values for vaccine-related parameters for HA-MRSA strains versus CA-MRSA strains, which may be an important aspect for future vaccine evaluations, in light of underlying genotypic differences.

**Table 1:** **Model parameters, with baseline values imported from**[23,24]

**Parameter**	**Symbol**	**Baseline value**
Total no. of patients	*N*	400
Fraction of patients colonized with CA-MRSA upon admission	$\lambda_{\mathrm{CC}}^{\mathrm{baseline}}$	0.03
Fraction of patients colonized with HA-MRSA upon admission	$\lambda_{\mathrm{CH}}^{\mathrm{baseline}}$	0.07
Fraction of patients infected with CA-MRSA upon admission	$\lambda_{\mathrm{IC}}^{\mathrm{baseline}}$	0.005
Fraction of patients infected with HA-MRSA upon admission	$\lambda_{\mathrm{IH}}^{\mathrm{baseline}}$	0.0017
Average length of stay susceptible patients	*1/γ*_S_	5 days
Average length of stay patients colonized with CA-MRSA	*1/γ*_CC_	5 days
Average length of stay patients colonized with HA-MRSA	*1/γ*_CH_	7 days
Average length of stay patients infected with CA-MRSA	*1/γ*_IC_	10 days
Average length of stay patients infected with HA-MRSA	*1/γ*_IH_	18 days
CA-MRSA colonized-to-susceptible patient effective transmission rate	$\beta_{\mathrm{CC}}^{\mathrm{baseline}}$	0.36 day^-1^
HA-MRSA colonized-to-susceptible patient effective transmission rate	$\beta_{\mathrm{CH}}^{\mathrm{baseline}}$	0.27 day^-1^
CA-MRSA infected-to-susceptible patient effective transmission rate	$\beta_{\mathrm{IC}}^{\mathrm{baseline}}$	0.09 day^-1^
HA-MRSA infected-to-susceptible patient effective transmission rate	$\beta_{\mathrm{IH}}^{\mathrm{baseline}}$	0.07 day^-1^
Infection rate in CA-MRSA colonized patients	φ_C_	0.1 γ_CC_
		(10% per day of hospital stay)
Infection rate in HA-MRSA colonized patients	φ_H_	0.1 γ_CH_
		(10% per day of hospital stay)
Death rate of CA-MRSA infected patients	δ_C_	0.033 γ_IC_
		(3.3% per day of hospital stay)
Death rate of HA-MRSA infected patients	δ_H_	0.2 γ_IH_
		(20% per day of hospital stay)
Cure rate of CA-MRSA infected patients	τ_C_	0.967 γ_IC_
		(96.7% per day of hospital stay)
Cure rate of HA-MRSA infected patients	τ_H_	0.8 γ_IH_
		(80% per day of hospital stay)

In the analyses presented here, for the time being we have considered identical vaccine-related parameters for both HA-MRSA and CA-MRSA so as to reduce the number of possible combinations of parameters.

The model’s setting is a large tertiary hospital in the US, with a fixed capacity of 400 beds, and approximately 25000 admissions per year [23]. For simplicity and consistency, and in the absence of hospital occupancy data over time, we assumed that the hospital is fully occupied at all times, throughout our simulations. We consequently varied the daily number of admissions (typically 60–80 patients/day in our simulations) to ensure full hospital capacity. In this simplified framework, we hypothesized that patients are adequately vaccinated with a *S. aureus* vaccine before hospital admission (e.g. planned hospitalization, vaccination of high-risk individuals), in an optimal timeframe (e.g., few weeks) to yield a protective immune response for the duration of the hospital stay. Similar to the baseline case from the original paper [23], we assumed a constant in-flow of colonized patients into the hospital corresponding to 10% (fixed) of the daily admission numbers.

### Model structure

**Figure 1:**
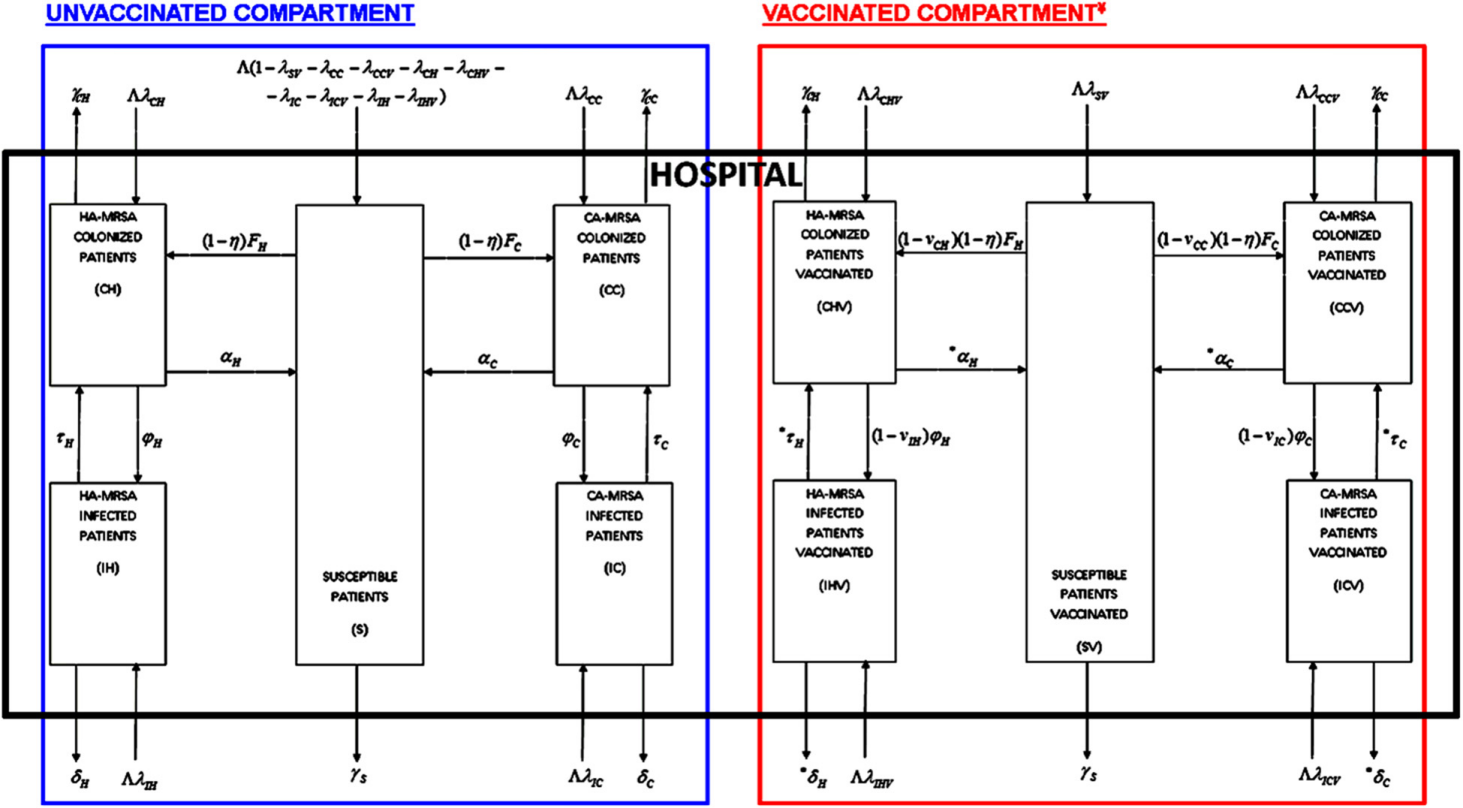
**Structure of dynamic transmission model with vaccination for MRSA infection in a hospital setting*.*** This model structure is following the model structure in [23]. Related parameter definitions and mathematical details can be found in Tables 1 and 2. This is a basic framework focused solely on transmission and infection at patient population level in a hospital setting, and not aiming to model dynamic feedback between hospital and community, patient history (pre- or post-hospitalization), etc. Patients are flowing into the hospital at a rate Λ defined as number of patients admitted per time unit (e.g., number of admissions per day), with corresponding fractions denoted by λ flowing in each of the Susceptible/Colonized (CA-MRSA/HA-MRSA)/Infected (CA-MRSA/HA-MRSA) states, unvaccinated and vaccinated, respectively, upon admission. We assume that vaccination takes place adequately prior to hospital admission, so that patients can mount a protective immune response; we do not consider in-hospital vaccination of current patients, under the assumption that the duration of the hospital stay is likely too short to enable a significant vaccine-induced immune response. We also do not consider vaccine protection waning for vaccinated patients while in the hospital, assuming the vaccine effects last at least for the duration of the current hospital stay. ^¥^ “*” indicates additional potential benefits of vaccination that can be considered in this type of modelling framework: (1) potential faster clearance in the vaccinated patients; (2) potential faster recovery from infection in the vaccinated patients (milder infections); (3) lower death rates in the infected vaccinated patients (milder infections). In the analyses performed here, we did not consider such enhanced vaccination effects, and all the related parameters are similar in the Unvaccinated and Vaccinated model components. The possibility of a vaccine impacting colonization [26-30] is taken into account as a potential reduction in the force of infection.

The detailed model structure including a hypothetical *S. aureus* vaccination component is shown in Figure 1, resulting in a highly versatile modelling framework, with enhanced options for conducting differential analyses. We employ a dynamic transmission model with two components, “Unvaccinated” and “Vaccinated”, respectively. Each component contains five distinctive states: susceptible (*S*) patients, patients colonized with HA-MRSA (*CH* patients), patients colonized with CA-MRSA (*CC* patients), patients infected with HA-MRSA (*IH* patients) or infected with CA-MRSA (*IC* patients). The additional “V” notation here is used for the corresponding vaccinated states. Patients are vaccinated (*V*) before being hospitalized and enter directly into the “vaccinated” compartment.

In Figure 1, the arrows indicate the inflows and outflows for each of the five states in the two model components, with their corresponding rates. The rate of hospital admissions is Λ, with corresponding fractions of patients admitted with CA-MRSA colonization, CA-MRSA infection, HA-MRSA colonization, and HA-MRSA infection denoted by *λ*_*CC(V)*_, *λ*_*IC(V)*_, *λ*_*CH(V)*_, and *λ*_*IH(V)*_, respectively (unvaccinated/vaccinated)*. λ*_*SV*_ represents the fraction of susceptible patients who received the vaccine prior to hospital admission. The mean length of hospital stay is defined as 1/*γ*_*S*,_ 1/*γ*_*C*_, and 1/*γ*_*H*_, for susceptible patients, patients colonized with CA-MRSA, and patients colonized with HA-MRSA, respectively.

For both the “Unvaccinated” and “Vaccinated” compartments here, the forces of infection (e.g., the per susceptible risk of infection) for HA-MRSA (*F*_*H*_) and CA-MRSA (*F*_*C*_), respectively, are governed by the following expressions:

$$F_{H}=\beta_{\mathrm{CH}}\left(\mathrm{CH}+\mathrm{CHV}\right)/N+\beta_{\mathrm{IH}}\left(\mathrm{IH}+\mathrm{IHV}\right)/N$$

$$F_{C}=\beta_{\mathrm{CC}}\left(\mathrm{CC}+\mathrm{CCV}\right)/N+\beta_{\mathrm{IC}}\;\left(\mathrm{IC}+\mathrm{ICV}\right)/N$$

Here, β_CC_, β_*IC*_, β_*CH*_, and β_*IH*_ denote the effective transmission rates to susceptible patients from patients with CA-MRSA colonization, CA-MRSA infection, HA-MRSA colonization, or HA-MRSA infection, respectively. *CC(V)*, *IC(V)*, *CH(V)*, and *IH(V)* denote the number of patients with CA-MRSA colonization, CA-MRSA infection, HA-MRSA colonization, or HA-MRSA infection, unvaccinated and vaccinated, respectively *N* denotes the total hospital patient population. Transmission within the hospital can occur as a result of direct contact with patients or contaminated fomites and Health Care Workers (HCW).

In this context, the HA- and CA-MRSA colonization rates of susceptible unvaccinated (*S*) patients are given by: (1-*η*)*F*_*H*_ and (1-*η*)*F*_*C*_, respectively, where *η* denotes the hospital hygiene compliance level. For vaccinated susceptible (*SV*) patients, we can also include the potential for vaccine efficacy against carriage acquisition, *ν*_*CC*_ and *ν*_*CH*_ - and thus the corresponding colonization terms can be amended as follows (1-*ν*_*CH*_)(1-*η*)*F*_*H*_ and (1-*ν*_*CC*_)(1-*η*)*F*_*C*_*,* respectively.

The infection rates in colonized patients with CA-MRSA and patients with HA-MRSA are *φ*_*C*_ and *φ*_*H*_, respectively. For the vaccinated population, we consider the vaccine efficacy against infection (*ν*_*IC*_ and *ν*_*IH*_) by introducing reduction factors in the corresponding infection rates as (1- *ν*_*IH*_) *φ*_*H*_ and (1- *ν*_*IC*_) *φ*_*C*_, respectively.

As a starting point, we considered similar vaccine efficacy against carriage or infection for both CA-MRSA and HA-MRSA, i.e. *ν*_*CC*_ = *ν*_*CH*_ = *ν*_*C*_ and *ν*_*IC*_ = *ν*_*IH*_ = *ν*_*I*_.

The cure rates of patients with CA-MRSA infection and HA-MRSA infection are *τ*_*C*_ and *τ*_*H*_, while the death rates of these patients are *δ*_*C*_ and *δ*_*H*_, respectively. The decolonization rates of patients with CA-MRSA colonization and HA-MRSA colonization are *α*_*C*_ and *α*_*H*_, respectively. The mathematical model formulation is standard for mechanistic deterministic compartmental models of disease spread in mathematical epidemiology, based on the law of mass action and resulting in a characteristic set of non-linear ordinary differential equations [31] For each of the ten distinctive model states illustrated by the different compartments in Figure 1, the rate of change over time equals the specific inflow minus the corresponding outflow, characterized by the rates indicated on the corresponding arrows in the model schematic in Figure 1, with related definitions and mathematical details given in Tables 1 and 2. The total hospital patient population *N* here is maintained constant at full hospital occupancy (*N* = 400 patients) by varying the hospital admission rate Λ so that the total mass balance is properly closed.

**Table 2:** Mathematical representation of different control strategies and related parameters in the current modelling framework

	**Unvaccinated**	**Vaccinated**
**Colonization rate CA-MRSA**	(1 - *η*)*F*_*C*_, 0 ≤ *η* ≤ 1	(1 - *ν*_*CC*_)(1 - *η*)*F*_*C*_, 0 ≤ *ν*_*CC*_ ≤ 1
		*ν*_*CC*_: **Vaccine efficacy against CA-MRSA colonization**
	*η*: **Hygiene compliance parameter**	
	*F*_*C*_ = *β*_*CC*_(*CC* + *CCV*)/*N* + *β*_*IC*_(*IC* + *ICV*)/*N*	
	$$\beta_{\mathrm{CC}}=\beta_{\mathrm{CC}}^{\mathrm{baseline}}-s\left(\beta_{\mathrm{CC}}^{\mathrm{baseline}}-\beta_{\mathrm{IC}}^{\mathrm{baseline}}\right),\;0\leq s\leq 1$$	
	$$\beta_{\mathrm{IC}}=\beta_{\mathrm{IC}}^{\mathrm{baseline}}$$	
	*s*: **Screening efficacy parameter**	
**Colonization rate HA-MRSA**	(1 - *η*)*F*_*H*_, 0 ≤ *η* ≤ 1	(1 - *ν*_*CH*_)(1 - *η*)*F*_*H*_, 0 ≤ *ν*_*CH*_ ≤ 1
		*ν*_*CH*_: **Vaccine efficacy against HA-MRSA colonization**
	*F*_*H*_ = *β*_*CH*_(*CH* + *CHV*)/*N* + *β*_*IH*_(*IH* + *IHV*)/*N*	
	$$\beta_{\mathrm{CH}}=\beta_{\mathrm{CH}}^{\mathrm{baseline}}-s\left(\beta_{\mathrm{CH}}^{\mathrm{baseline}}-\beta_{\mathrm{IH}}^{\mathrm{baseline}}\right),\;0\leq s\leq 1$$	
	$$\beta_{\mathrm{IH}}=\beta_{\mathrm{IH}}^{\mathrm{baseline}}$$	
**Decolonization rate CA-MRSA**	*α*_*C*_	$\alpha_{{}_{C}}^{*}$
	$$\alpha_{{}_{C}}^{*}=\alpha_{C}$$	
	*α*_*C*_ = *dγ*_*CC*_, 0 ≤ *d* ≤ 1	
	*d*: **Decolonization efficacy parameter**	
**Decolonization rate HA-MRSA**	*α*_*H*_	$\alpha_{{}_{H}}^{*}$
	$$\alpha_{{}_{H}}^{*}=\alpha_{H}$$	
	*α*_*H*_ = *dγ*_*CH*_, 0 ≤ *d* ≤ 1	
**Rate of progression to CA-MRSA infection after colonization**	*φ*_*C*_	(1 - *ν*_*IC*_)*φ*_*C*_, 0 ≤ *ν*_*IC*_ ≤ 1
		*ν*_*IC*_: **Vaccine efficacy against CA-MRSA infection**
**Rate of progression to HA-MRSA infection after colonization**	*φ*_*H*_	(1 - *ν*_*IH*_) *φ*_*H*_, 0 ≤ *ν*_*IH*_ ≤ 1
		*ν*_*IH*_: **Vaccine efficacy against HA-MRSA infection**
**Vaccination prior to hospital admission**	Fraction of vaccinated susceptible flowing into the hospital:	
	$$\lambda_{\mathrm{SV}}=c\left(1-\lambda_{\mathrm{CC}}^{\mathrm{baseline}}-\lambda_{\mathrm{CH}}^{\mathrm{baseline}}-\lambda_{\mathrm{IC}}^{\mathrm{baseline}}-\lambda_{\mathrm{IH}}^{\mathrm{baseline}}\right)$$	
	Fraction of vaccinated colonized CA-MRSA flowing into the hospital	
	$$\lambda_{\mathrm{CCV}}=c\lambda_{\mathrm{CC}}^{\mathrm{baseline}}$$	
	Fraction of vaccinated colonized HA-MRSA flowing into the hospital:	
	$$\lambda_{\mathrm{CHV}}=c\lambda_{\mathrm{CH}}^{\mathrm{baseline}}$$	
	Fraction of vaccinated infected CA-MRSA flowing into the hospital:	
	$$\lambda_{\mathrm{ICV}}=c\lambda_{\mathrm{IC}}^{\mathrm{baseline}}$$	
	Fraction of vaccinated infected HA-MRSA flowing into the hospital:	
	$$\lambda_{\mathrm{IHV}}=c\lambda_{\mathrm{IH}}^{\mathrm{baseline}}$$	
	Fraction of unvaccinated colonized CA-MRSA flowing into the hospital:	
	$$\lambda_{\mathrm{CC}}=\left(1-c\right)\lambda_{\mathrm{CC}}^{\mathrm{baseline}}$$	
	Fraction of unvaccinated colonized HA-MRSA flowing into the hospital:	
	$$\lambda_{\mathrm{CH}}=\left(1-c\right)\lambda_{\mathrm{CH}}^{\mathrm{baseline}}$$	
	Fraction of unvaccinated infected CA-MRSA flowing into the hospital:	
	$$\lambda_{\mathrm{IC}}=\left(1-c\right)\lambda_{\mathrm{IC}}^{\mathrm{baseline}}$$	
	Fraction of unvaccinated infected HA-MRSA flowing into the hospital:	
	$$\lambda_{\mathrm{IH}}=\left(1-c\right)\lambda_{\mathrm{IH}}^{\mathrm{baseline}}$$	
	*c*: **Vaccine coverage parameter,** 0 ≤ *c* ≤ 1	

### Model parameters

In this analysis, we are interested in outcomes while varying the following input parameters outlined in Table 2:

1. Hospital hygiene compliance with measures aimed at reducing MRSA transmission (e.g. hand hygiene, wearing gloves), governed by the hygiene compliance model parameter η. Compliance level may vary between zero (corresponding to 0% compliance, η = 0) and one (corresponding to 100% compliance, η = 1).

2. Hospital adherence and compliance to patients’ screening and isolation for MRSA colonization: finding the carriers and placing them on contact precautions within the hospital, governed by the screening efficacy model parameter *s*. It may vary between 0% (*s =* 0): no MRSA carrier is placed on contact precaution and 100% (*s =* 1): all MRSA carriers are placed on the same contact precautions as the MRSA-infected patients.

3. Hospital decolonization of colonized patients: identifying the carriers and applying decolonization regimens within the hospital, governed by the decolonization efficacy model parameter *d*. It may vary between 0% (*d*_*=*0_): no successful decolonization, and 100% (*d*_=1_): 100% of patients decolonized daily return to susceptible status. Various combinations of control strategies 1–3 can be considered to mimic implementation of bundle measures in the hospital.

4. Vaccine coverage: fraction of admitted patients who got vaccinated prior to admission to the hospital, governed by the vaccine coverage model parameter *c*. It can be varied between 0% (*c*_=0_) and 100% (*c*_=1_).

5. Vaccine efficacy against colonization: vaccine-induced protection against carriage acquisition (reduced risk of getting colonized once vaccinated), governed by the vaccine efficacy model parameters *ν*_*CC*_ = *ν*_*CH*_). This can vary between 0% (same risk of carriage acquisition in unvaccinated and vaccinated patients, *ν*_*CC*_ = *ν*_*CH*_ = 0) and 100% (none of the vaccinated patients become colonized, in the hospital *ν*_*CC*_ = *ν*_*CH*_ = 1).

6. Vaccine efficacy against infection if colonized: vaccine-induced protection against developing MRSA infection in MRSA-colonized vaccinated patients (reduced risk of developing MRSA infection in MRSA vaccinated colonized patients), governed by the vaccine efficacy model parameter *ν*_*IC*_ = *ν*_*IH*_). This can vary between 0% (vaccinated MRSA-colonized patients develop MRSA infection at the same rate as non-vaccinated MRSA-colonized patients, *ν*_*IC*_ = *ν*_*IH*_ = 0) and 100% (no vaccinated MRSA-colonized patients develop infection, *ν*_*IC*_ = *ν*_*IH*_ = 1).

In this paper, we are primarily interested in model-based projections of MRSA infection reduction in a hospital setting following implementation of different hygiene prevention measures and potential *S. aureus* vaccination scenarios. This can be achieved by varying the values of the six model parameters between 0% and 100% accordingly. A certain level of compliance with hygiene prevention measures (% hand hygiene +/-% screening +/-% decolonization) will presumably result in a consequent projected reduction in the number of MRSA infections. Upon potential implementation of vaccination, in addition to hygiene prevention measures, a further reduction in the rate of infection is conceivable. The projected magnitude of this additional reduction will depend on the existing hygiene prevention measures, and compliance to them. An intuitive argument could be made that if compliance with hygiene prevention measures is sufficiently high, there may be less potential for additional reduction of MRSA infection via vaccination with a *S. aureus* vaccine; from both a public health as well as a vaccine development perspective, however, more evidence-based answers and estimates are needed.

In-line with the primary purpose of this work, for all the outcomes presented here, with the exception of the six parameters described above, all the other model parameters were kept fixed at the baseline values from the original publications [23,24], which we employ here at face value. These baseline parameter values, as described in [23,24], are summarized here in Table 1 for completeness and clarity. Although various other sensitivity analyses could be carried out, in order to maintain tractability, in the present analysis we chose to restrict the scope and focus on the model parameters controlling the different interventions. Sensitivity analyses with respect to other model input parameters are presented in the original paper by D’Agata *et al.*[23], and more technical and mathematical details are discussed in the paper published by Webb *et al.*[24]. All our numerical simulations are carried out in MATLAB R2010b The MathWorks, Inc., Natick, Massachusetts, United States.

## Results

All the results presented here are at steady-state, as transient regimes are short-lived i.e. a few months (see also Figure 2 in [23]) and not of main interest for longer term infection control policies in the hospital.

**Figure 2:**
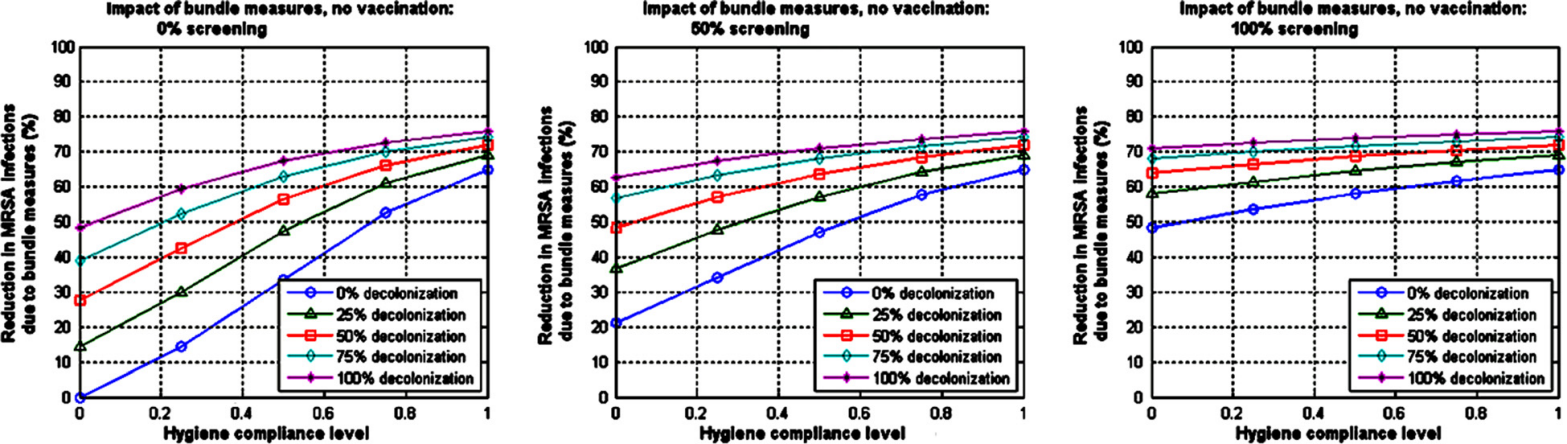
**Illustration of model-projected impact of bundle measures only (no vaccination) on MRSA infection prevalence reduction.** All results shown here are at steady-state.

The key terms employed in this section are summarized for clarity in Table 3.The impact of hygiene prevention measures on MRSA infection, as function of compliance, is illustrated in Figure 2. All the percentage reductions in MRSA infection prevalence shown in this figure were computed relative to a base case corresponding to no hygiene prevention measures in place. With 50% screening compliance and 50% efficacy of decolonization, a model-projected reduction of 48% can be achieved even at 0% hygiene compliance levels; this reduction can go up to 72% under similar conditions, assuming 100% hygiene compliance levels. At 100% screening compliance and 50% efficacy of decolonization, a reduction of 64% is projected by the model at 0% hygiene compliance level. The maximum reduction projected by this model in a best case scenario (100% screening compliance and 100% efficacy of decolonization) is 76% (Figure 2).

**Table 3:** Definition of key terms employed throughout the analyses

**Term**	**Definition**
**Hygiene compliance level**	Hospital compliance with hand washing, wearing gloves, etc., varying between 0 (corresponding to hygiene compliance parameter *η* = 0) and 1 (corresponding to hygiene compliance parameter *η* = 1).
**Screening**	Identify the carriers and place them on contact precautions within the hospital. “x% screening” interpretation here: hospital compliance to finding and placing carriers on contact precautions; varies between 0% (corresponding to screening efficacy parameter *s* = 0): no MRSA carrier is placed on contact precaution and 100% (corresponding to screening efficacy parameter *s* = 1): all MRSA carriers are placed on the same contact precautions as the MRSA-infected patients.
**Decolonization**	Identify the carriers and apply decolonization regimens within the hospital. “x% decolonization” interpretation here: actual efficacy of decolonization protocol, varying between 0% (corresponding to decolonization efficacy parameter *d* = 0): no successful decolonization, and 100% (corresponding to decolonization efficacy parameter *d* = 1): 100% of patients decolonized daily return to susceptible status.
**Vaccine efficacy against colonization**	Vaccine-induced protection against carriage acquisition (reduced risk of colonization once vaccinated); can be varied between 0% (corresponding to vaccine efficacy parameter *ν*_*CC*_ = *ν*_*CH*_ = 0) and 100% (corresponding to vaccine efficacy parameter *ν*_*CC*_ = *ν*_*CH*_ = 1). Here we assumed for simplicity that *ν*_*CC*_ = *ν*_*CH*_.
**Vaccine efficacy against infection if colonized**	Vaccine-induced protection against developing actual MRSA infection in MRSA-colonized patients (lower risk of developing MRSA infection in MRSA vaccinated colonized patients); can be varied between 0% (corresponding to vaccine efficacy parameter *ν*_*IC*_ = *ν*_*IH*_ = 0) and 100% (corresponding to vaccine efficacy parameter *ν*_*IC*_ = *ν*_*IH*_ = 1) . Here we assumed for simplicity that *ν*_*IC*_ = *ν*_*IH*_.
**Vaccine coverage**	Fraction of admitted patients who got vaccinated prior to admission to the hospital. “x% vaccine coverage” interpretation here: x% of the patients admitted daily into the hospital have been vaccinated prior to admission; can be varied between 0% (corresponding to vaccine coverage parameter *c* = 0) and 100% (corresponding to vaccine coverage parameter *c* = 1).

**Figure 3:**
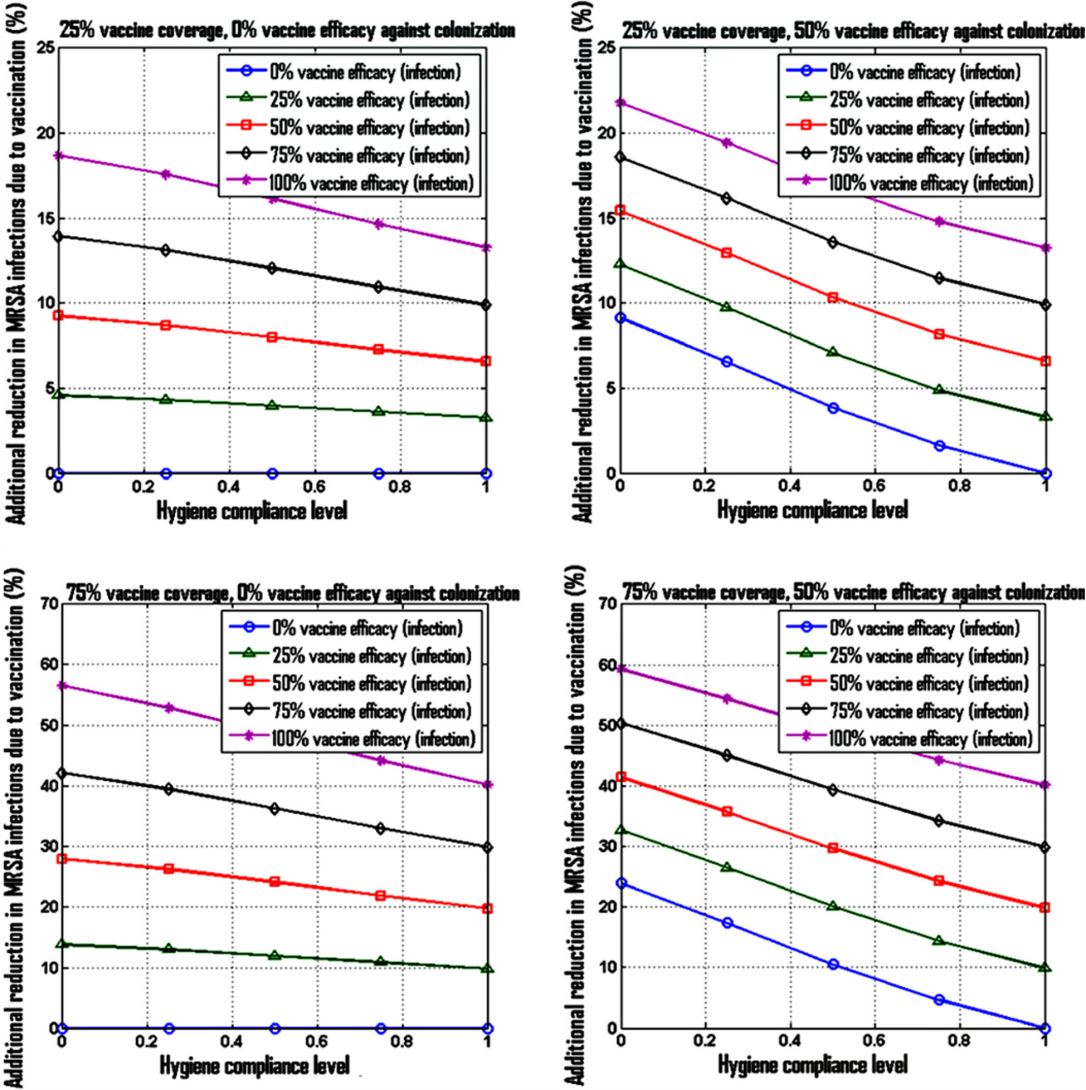
**Illustration of simulated additional reduction in MRSA infection via vaccination on top of bundle measures.** Model-projected relative (%) reduction in MRSA infection prevalence due to vaccination on top of bundle measures for an average case scenario with 50% decolonization and 50% screening. The top plots correspond to a 25% vaccine coverage scenario and the bottom plots to a 75% vaccine coverage scenario. The plots on the left were generated assuming that a potential vaccine would have no impact on colonization, while the corresponding plots on the right illustrate potential added benefits of a vaccine that would be 50% efficient at preventing colonization. All results shown are at steady-state.

Figure 3 illustrates the relative (%) additional reduction in MRSA infection prevalence due to *S. aureus* vaccination, as a function of the hygiene compliance levels, assuming hygiene measures are in place, for a vaccine coverage fixed at 25% (Figure 3, top panels) and 75% (Figure 3, bottom panels) respectively. Each percentage reduction in MRSA infection prevalence shown in this figure was computed relative to a corresponding base case without vaccination. Hence, the reductions reported in this figure reflect the potential added impact of vaccination on top of other existing preventative measures, for various case scenarios.

**Figure 4:**
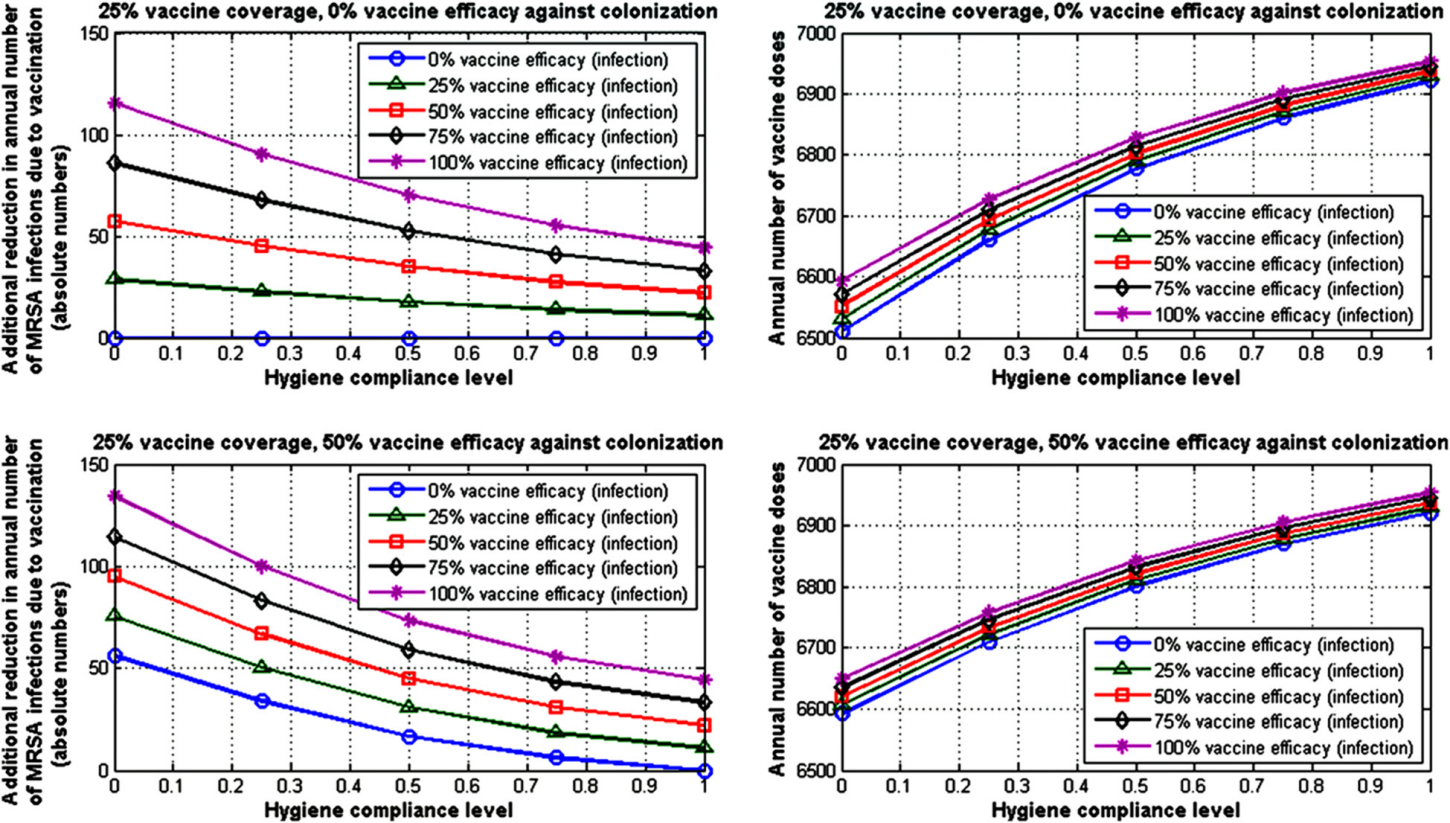
**Model-based projections, 25% vaccine coverage: annual number of cases averted and corresponding number of doses.** Left panel: model-based projected reduction in the annual number of MRSA infections due to vaccination at 25% vaccine coverage on top of bundle measures, as a function of the hygiene compliance level, for an average case scenario with 50% decolonization and 50% screening. Right panel: annual number of vaccine doses necessary to achieve the corresponding levels of MRSA infection reduction, assuming one dose per patient. All results are shown at steady-state. The slight variations in the number of doses here for the same level of vaccine coverage reflect the corresponding differences in the number of daily admissions, which is allowed to vary in each instance to ensure full hospital occupancy at all times.

**Figure 5:**
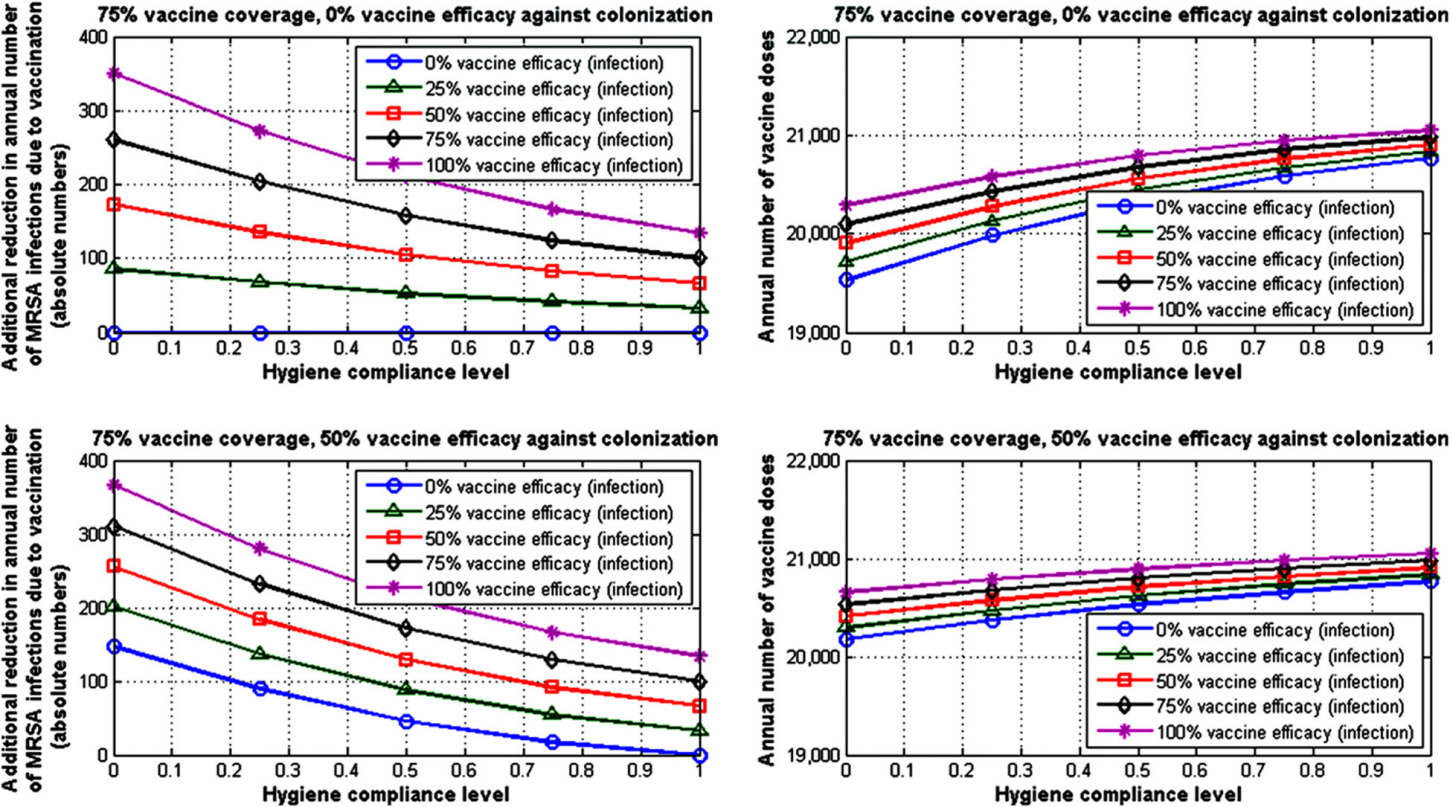
**Model-based projections, 75% vaccine coverage: annual number of cases averted and corresponding number of doses.** Left panel: model-based projected reduction in the annual number of MRSA infections due to vaccination at 75% vaccine coverage on top of bundle measures, as a function of the hygiene compliance level for an average case scenario with 50% decolonization and 50% screening. Right panel: annual number of vaccine doses necessary to achieve the corresponding levels of MRSA infection reduction, assuming one dose per patient. All results are shown at steady-state. The slight variations in the number of doses here for the same level of vaccine coverage reflect the corresponding differences in the number of daily admissions, which is allowed to vary in each instance to ensure full hospital occupancy at all times.

We would like to note here that due to the inherent high-dimensional nature of this analysis, based on varying six model parameters, there are many different possibilities for visualizing and reporting the outcomes. The figures shown here, while non-exhaustive, are intended to reflect results in-line with the primary scope of this work.As an illustrative example, consider for instance an average case scenario for preventative measures in place, with 50% decolonization efficacy, 50% screening compliance, and 50% hygiene compliance level. In such a case, for a vaccination case scenario with 25% vaccine coverage and 75% vaccine efficacy against infection, the model-estimated additional reduction in MRSA infection prevalence due to vaccination is 12% (Figure 3 [top left]), assuming 0% vaccine efficacy against colonization. Alternatively, for the same average case, with vaccine coverage of 75%, and vaccine efficacy against infection of 75%, the model-estimated additional reduction in MRSA infections due to vaccination is 37% (Figure 3 [bottom left]), assuming 0% vaccine efficacy against colonization. Higher additional reductions could be achieved if the vaccine had protective effects against carriage acquisition as well (Figure 3 [top right] and [bottom right]).Figures 4 and 5 show the annual number of vaccine doses in each of these case scenarios (right panels) and the corresponding number of MRSA infections averted (left panels) due to vaccination, assuming one vaccine dose per patient. In our “average” preventative measure case discussed above, for a vaccination case scenario with 25% vaccine coverage and 75% vaccine efficacy against infection, the model estimates that at 52 additional cases of MRSA infection could be averted annually in a hospital setting with 6815 doses of vaccine (Figure 4 [top]), assuming 0% vaccine efficacy against colonization. Alternatively, with vaccine coverage of 75%, and vaccine efficacy against infection still at 75%, the model estimates that 158 additional cases of MRSA infection could be averted annually in a hospital setting with 20680 doses of vaccine (Figure 5 [top]), assuming 0% vaccine efficacy against colonization. Additional benefits that could be obtained if a potential vaccine had an impact on colonization as well are illustrated in the corresponding plots in Figure 4 [bottom] and Figure 5 [bottom], for a case scenario where a vaccine would have 50% efficacy against colonization.

## Discussion

In this paper, we explore for the first time whether hypothetical *S. aureus* vaccination in addition to other preventative measures may have the potential to further reduce MRSA infection in a given hospital setting and we attempt to provide estimates of the potential magnitude of such reductions under various scenarios. To this end, we employ a versatile dynamic transmission modelling framework. Model-based projections indicate that even with implementation of other hygiene prevention measures, *S. aureus* vaccination could potentially provide additional reduction of MRSA infection in a hospital setting. Vaccine coverage and vaccine efficacy are key factors that would impact the magnitude of this reduction. To our knowledge, this is the first study to assess potential of a *S. aureus* vaccine to yield additional MRSA infection reduction in a hospital setting, on top of other preventative measures that already proved efficient.

Model-based simulations illustrate how different levels of reduction of MRSA infection can be achieved at the hospital level with various combinations of interventions such as hospital hygiene, decolonization and contact precautions. These interventions have been shown to be effective in decreasing the spread of MRSA in hospitals [10-15,18], but their impact is variable, depending on the level of compliance. Non-compliance with hand hygiene is recognized as the most important modifiable cause of healthcare-acquired infections [32,33]. Despite existing guidelines, rates of compliance with hand hygiene recommendations remain low in hospitals. The Center for Disease Control and Prevention reported that among HCW, the national average rate of compliance with recommended hand hygiene procedures in the US was approximately 40% in 2002 [32]. However, higher compliance with hygiene recommendations is reported in different hospital settings, reaching for instance 90% in the Duke Hospital network in 2010 [34]. According to a recent comprehensive review of literature published as a Cochrane Review [35], the quality of intervention studies intended to evaluate and to improve hand hygiene compliance remains low.

Robicsek *et al.*[14] reported that the implementation of universal MRSA surveillance (about 90%, screening) plus decolonization and isolation in a 3-hospital network (with about 850 beds) led to a 69.6% reduction in the number of MRSA infections compared to baseline (prior to introduction of active surveillance) [14]. Using similar measures, Jain and colleagues [18] reported a decrease of 62% in the number of healthcare-associated MRSA infections in ICUs belonging to Veterans Affairs hospitals. Our model projections estimate reductions in MRSA infection prevalence of 60-75% under related conditions.

However, patient protection against MRSA infection based on such interventions only is likely to be limited to the hospitalization period and up to 30 days after hospital discharge [14], while a vaccine may potentially provide longer term protection (e.g. several months [36]). This is an important aspect since patients who are more susceptible to HA-MRSA and CA-MRSA hospital infections [37-39] may also require repeated episodes of hospitalization [40], which would presumably be reduced through the implementation of MRSA preventative measures. Recently, Huang *et al.*[40] reported the results of a retrospective cohort study evaluating the risk of MRSA infection, hospitalization and death after hospital discharge among high-risk patients who had been newly identified as harbouring MRSA. Between January 1991 and December 2003, 591 new MRSA carriers were identified: 23% colonized and 77% infected, at the time of detection. In the year following the identification of the carrier state, 196 patients developed 317 MRSA infections. Of these cases of MRSA infections, 26% involved bacteraemia and 17% led to MRSA-attributable death [40].

Here, we emphasize the fact that the model considered in this paper is for MRSA only, focusing on the reduction in MRSA infection in a hospital setting, and does not include MSSA. MSSA continues to represent a substantial proportion of all *S. aureus* infections in hospitals [1], and future expansions of the model to include MSSA should be considered if similar MSSA hospital transmission data become available. One of the reasons for building a vaccination component starting from an existing versatile model [23] was the fact that baseline values for the model parameters were coherently provided in a given hospital setting. Generally, model parameters (such as transmission and progression rates) need to be estimated by calibrating the model against data. For such models to be useful in practice, the level of model complexity needs to be supported by available data. Here, we did not have data available for MSSA in this hospital setting. Also, following the original model in [23,24], in this simplified modeling framework, dual CA-MRSA/HA-MRSA colonization is not explicitly considered. Information/data about co-colonization with CA-MRSA and HA-MRSA is currently largely missing, as most studies tend to look at colonization separately. Co-existence in this modeling framework is enabled via adequate steady influxes of correspondingly colonized/infected patients into the hospital. More discussions can be found in [23,24].

We also emphasize that in practice, model-based projections may be setting/hospital-dependent, as the input parameters for the underlying baseline model may vary from one hospital setting to another. In this analysis, we employed at face-value published baseline values [23,24] for the model parameters governing infection transmission and spread in a given hospital setting. Such estimates, which are key to any subsequent model outcome, can be challenged and should ideally be obtained based on model adaptation and calibration to specific hospital settings. Rationale was provided and documented by D’Agata et al., [23], and Webb *et al.,*[24], and here we simply accepted their framework as plausible in the given hospital setting in US and used it as a starting point for the type of analyses we were primarily interested in. To ascertain actual ranges and variability for the different input parameters (e.g., inflow of colonized/infected patients into the hospital, transmission/progression rates) so as to guide sensitivity analyses, further studies are necessary to collect and collate data from different hospital settings reflecting inherent heterogeneities (e.g., different geographical location, different type of hospital, patient population/community served).

As expected, assumed vaccine coverage has a significant impact on the model outcomes. We varied the vaccine coverage parameter between 0 and 100%, and we showed here results corresponding to low (25%) and high (75%) vaccine coverage, respectively, for illustrative purposes. The actual levels of coverage attainable in practice are likely to depend greatly on the type of vaccination strategy (e.g., community vaccination, vaccination of planned intervention patients) as well as hospital type (e.g., general vs. specialty). A significant percentage of hospitalizations in the US are due to interventions [41], most of which are planned [42]. This percentage is typically higher in specialty hospitals [43].Vaccinating patients prior to hospital admission may theoretically provide adequate time to elicit a protective immune response [44]. In a recent Phase IIB/III clinical trial, patients were vaccinated with a *S. aureus* candidate vaccine 14–60 days before planned cardiovascular surgery [45].

The work we present here was not intended as exhaustive or definitive, but rather a starting point. Our goals were to propose a versatile and tractable modelling and simulation framework for evaluating the impact of combined interventions on reducing MRSA infection at the hospital level and illustrate it with concrete results we found relevant in the context as a proof of concept. The proposed framework can serve further for additional analyses (e.g., cost-effectiveness) and model adaptations (e.g., other preventative measures such as screening at admission), as well as potential comparison with other baseline models (e.g., different model parameters, simplified/other approaches, etc.).

Pending data availability, further expansions of this type of modelling framework could be considered to include a dynamic exchange and double feedback between the community and the hospital setting.

Finally, we want to acknowledge that the mechanistic deterministic modelling approach we employed here, while appealing because of its built-in tractability, is limited in the sense that everything is assumed as an average at a patient population/hospital level, with no explicit account for various sources of heterogeneity, variability and uncertainty. Particularly in smaller size hospitals, such an approach may not be ideally-suited, and alternatives such as stochastic and/or individual-based models could be considered.

## Conclusions

We have shown here how adequate *S. aureus* vaccination, prior to hospital admission, has the potential to further reduce burden of MRSA infection in the hospital setting, with magnitude of additional reduction varying depending on the level of other hygiene prevention measures in place. Current prevention measures such as hand hygiene are effective at reducing MRSA infection, but compliance with these measures is critical. Therefore, it is important that these practices continue to be promoted and implemented by hospitals and healthcare workers.

## Abbreviations

MRSA: Methicillin-resistant *S. aureus*; HA-MRSA: Healthcare-associated MRSA; CA-MRSA: Community-associated MRSA; VRE: Vancomycin-resistant enterococcus; ICUs: Intensive care units; MSSA: Methicillin sensitive *S. aureus*; HCW: Health care workers; CH patients: Patients colonized with HA-MRSA; CC patients: Patients colonized with CA-MRSA; IH patients: Patients infected with HA-MRSA; IC patients: Patients infected with CA-MRSA; FOI: Force of infection; S patients: Susceptible patients; SV: Vaccinated susceptible patients.

## Competing interests

AC, CH and TVE declare they are employed by GlaxoSmithKline group of companies and own stock options of GlaxoSmithKline.

## Authors’ contributions

CH designed the appropriate modelling framework, identified the data to support the model by extensive research of the literature, written the mathematical model with vaccination, implemented it in Matlab, devised the solution procedure and ran the computer simulations, devised various sensitivity analyses to address the driving questions, and provide specific answers. TVE was involved in interpretation of the modelling results. AC was involved in the design of the model and interpretation of the results. All authors read and approved the final manuscript.

## Pre-publication history

The pre-publication history for this paper can be accessed here:

http://www.biomedcentral.com/1471-2334/14/291/prepub
